# Nonlinear Association Between Body Roundness Index and Axial Spinal Pain in Middle‐Aged and Older Chinese Adults: A Nationwide Cross‐Sectional Study

**DOI:** 10.1155/prm/3187891

**Published:** 2026-04-01

**Authors:** Chao Mei, Zhanfeng Zhang, Jikang Min, Zengbing Xia, Wenlin Hu, Jianxiang Zhu

**Affiliations:** ^1^ Department of Endocrinology, The First People’s Hospital of Huzhou, Huzhou, 313000, Zhejiang, China, hz1y.com; ^2^ Department of Orthopedics, The First People’s Hospital of Huzhou, Huzhou, 313000, Zhejiang, China, hz1y.com

**Keywords:** axial spinal pain, body roundness index, CHARLS, cross-sectional study, older adults

## Abstract

**Background:**

Axial spinal pain is a major cause of disability in middle‐aged and older adults. The body roundness index (BRI) reflects central fat distribution, but its association with axial spinal pain is unclear.

**Methods:**

We analyzed cross‐sectional data from 10,689 adults aged ≥ 50 years from a national cohort in China. The association between BRI and axial spinal pain was examined using multivariable logistic regression, restricted cubic splines, subgroup analyses, and an evaluation of combined BRI and body mass index (BMI) categories.

**Results:**

Higher BRI was associated with increased odds of axial spinal pain (adjusted OR = 1.05 per unit; 95% CI 1.01–1.08), with the highest quartile showing 22% greater odds than the lowest (OR = 1.22; 95% CI 1.06–1.40). The association was nonlinear (threshold near BRI = 3.0) and modified by drinking status (*p* interaction = 0.007). The greatest odds were observed in individuals with both high BMI and high BRI (OR = 1.16; 95% CI 1.04–1.30).

**Conclusions:**

In this cross‐sectional study, elevated BRI was independently and nonlinearly associated with axial spinal pain, with stronger associations in drinkers and high‐BMI individuals. BRI shows promise for community health profiling, though longitudinal studies are needed to establish causality.

## 1. Introduction

Musculoskeletal pain, particularly axial spinal pain, is a leading cause of global disability, especially in middle‐aged and older adults [1]. Affecting mainly the lumbar and thoracic regions [2, 3], axial spinal pain contributes substantially to functional impairment, reduced quality of life, and greater healthcare utilization [4]. Low back or waist pain—a core component—affects an estimated 15%–30% of middle‐aged individuals, with prevalence increasing in older adults [5]. In China, axial spinal pain is common among older adults and poses an escalating public health challenge amid rapid population aging [6, 7].

Obesity, especially central adiposity, is an established risk factor for spinal pain through mechanical and biological mechanisms [8]. Mechanical effects include increased spinal loading, altered curvature, and accelerated degeneration of intervertebral discs and facet joints [9]. Biologically, visceral fat releases proinflammatory cytokines such as interleukin‐6 and tumor necrosis factor‐α, which can heighten pain perception and sustain chronicity [10]. Body mass index (BMI) is the most widely used anthropometric indicator, yet it cannot differentiate fat from muscle or capture fat distribution. Prior evidence on BMI and spinal pain is weak or inconsistent [11, 12], indicating that BMI may underestimate the impact of central adiposity.

The body roundness index (BRI), calculated from waist circumference (WC) and height, more accurately reflects body shape and central fat accumulation than BMI or WC alone [13]. BRI has demonstrated superior predictive ability for cardiometabolic risk, metabolic syndrome, and mortality [14–16]. However, its association with musculoskeletal health—particularly axial spinal pain—remains poorly understood. We therefore conducted a nationally representative, analytical cross‐sectional study using baseline data from the China Health and Retirement Longitudinal Study (CHARLS) to assess the relationship between BRI and axial spinal pain, explore variations across demographic, lifestyle, and health subgroups, and examine its combined effect with BMI.

## 2. Methods

### 2.1. Study Design and Population

We performed a cross‐sectional analysis using baseline data from the 2011 CHARLS, a nationally representative survey of Chinese adults aged ≥ 45 years. CHARLS employed a multistage, stratified, cluster sampling design to ensure representativeness across urban and rural areas and diverse socioeconomic strata. Data were collected through standardized, in‐home interviews and physical examinations conducted by trained personnel under rigorous quality control procedures.

**FIGURE 1:**
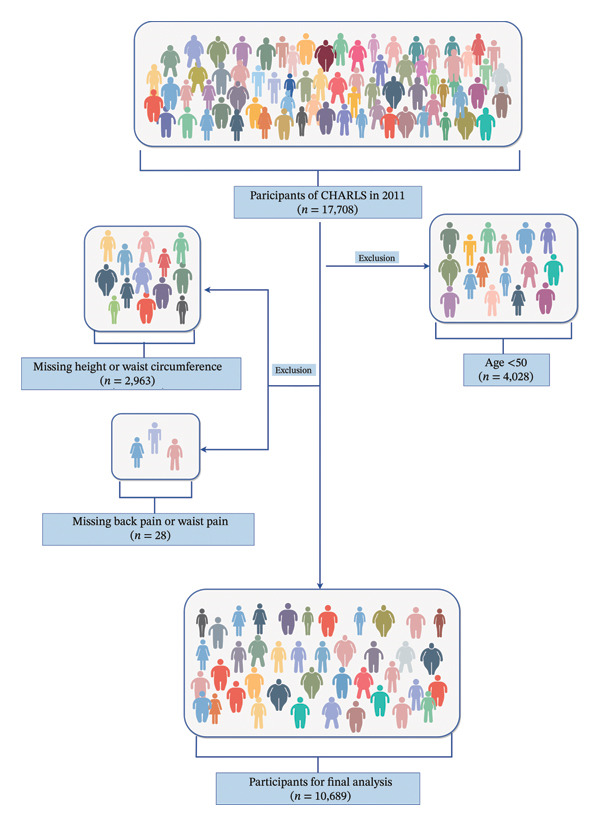
Participant flow diagram of the CHARLS 2011 study. A total of 17,708 participants were screened. After exclusions for age < 50, missing data on height, waist circumference, and missing pain data, 10,689 participants were included in the final analysis.

The original cohort comprised 17,708 participants. For this analysis, we excluded individuals aged < 50 years (*n* = 4028), those missing anthropometric measurements (height or WC; *n* = 2963), and those without pain assessment data (*n* = 28), resulting in a final analytic sample of 10,689 participants. The participant selection process is illustrated in Figure 1.

### 2.2. Ethical Approval and Consent to Participate

The CHARLS baseline survey received ethical approval from the Biomedical Ethics Review Committee of Peking University (Approval No. IRB00001052‐11014). All study procedures were conducted in strict accordance with the ethical principles of the Declaration of Helsinki, as well as relevant national and institutional regulations. Written informed consent was obtained from every participant prior to their inclusion in the study. The reporting of this cross‐sectional analysis follows the Strengthening the Reporting of Observational Studies in Epidemiology (STROBE) guidelines [17].

### 2.3. Assessment of Exposure: BRI

The primary exposure was the BRI, a novel anthropometric measure reflecting body fat distribution and central adiposity. BRI was calculated from height and WC using the following validated formula [13]:

(1) BRI=364.2365.5−×1−WC/2×π20.5×Height2.

Both height and WC were recorded in centimeters. WC was measured to the nearest 0.1 cm with a nonelastic tape placed horizontally at the midpoint between the lower edge of the rib cage and the upper border of the iliac crest (approximately at the umbilicus level). Measurements were taken at the end of a normal expiration with participants standing upright, feet together, and abdomen relaxed.

Standing height was measured without footwear using a calibrated stadiometer (Seca 213; Seca GmbH, Hamburg, Germany). Participants maintained an erect posture with the head positioned in the Frankfurt horizontal plane. All anthropometric measurements were obtained in duplicate by trained personnel following standardized protocols to ensure accuracy and reproducibility. If the two measurements differed by more than 0.5 cm, a third measurement was taken, and the mean of the two closest values was used for analysis.

### 2.4. Outcome Variable: Axial Spinal Pain

Axial spinal pain was assessed using the self‐reported pain questionnaire in CHARLS. Participants were first asked whether they had experienced any bodily pain. Those reporting pain were then asked to identify all affected anatomical sites.

For this analysis, axial spinal pain was defined as pain in at least one of two midline spinal regions: the back (thoracic region) or the waist (lumbar region). Pain reported in other locations—including the neck, head, shoulders, upper and lower limbs, and distal joints—was not considered axial spinal pain. The outcome variable was coded dichotomously (presence vs. absence of axial spinal pain).

### 2.5. Covariates

Covariate data were collected during the 2011 CHARLS baseline survey through standardized questionnaires, anthropometric measurements, and laboratory examinations.

Anthropometric variables included height (cm), WC (cm), and BMI, calculated as weight in kilograms divided by the square of height in meters. Demographic variables comprised age (years), sex (male or female), educational attainment (no formal schooling, elementary school, middle/high school, or college and above), marital status (married vs. unmarried/other), place of residence (rural or urban), health insurance coverage (yes or no), and current employment status (yes or no). Lifestyle factors included smoking status (never, former, and current) and alcohol consumption status (never, former, and current). Functional capacity was assessed using the instrumental activities of daily living (IADL) scale, covering tasks such as shopping, cooking, managing finances, and taking medications. Participants indicated whether health problems caused difficulty in performing each task; a “yes” response was classified as a limitation for that specific activity. The total number of limitations was summed to generate an overall IADL limitation score for each participant.

Information on chronic conditions, including hypertension and diabetes, was obtained via self‐reported physician diagnosis by asking: “Have you ever been diagnosed with [condition] by a doctor?”

### 2.6. Statistical Analysis

Continuous variables are presented as mean ± standard deviation (SD) for approximately normally distributed data or median (interquartile range, IQR) for skewed data, while categorical variables are expressed as frequency (percentage). Differences across BRI quartiles were evaluated using the Chi‐square test for categorical variables, one‐way analysis of variance (ANOVA) for normally distributed continuous variables, and the Kruskal–Wallis test for non‐normally distributed variables.

Associations between BRI and the prevalence of axial spinal pain were examined using logistic regression models. BRI was analyzed both as a continuous variable and in quartiles, using the lowest quartile as the reference category. Four hierarchical models were constructed: (1) unadjusted (crude) model; (2) adjusted for age and sex; (3) further adjusted for socioeconomic and lifestyle factors (education level, marital status, residential area, health insurance coverage, current employment status, smoking, and drinking status); and (4) additionally adjusted for health‐related covariates (depressive symptoms, hypertension, diabetes, and IADL disability). Linear trends across BRI quartiles were tested by entering the median value of each quartile as a continuous variable in the model.

To assess potential nonlinear associations between BRI and axial spinal pain, restricted cubic spline (RCS) regression models were fitted with three knots placed at the 10th, 50th, and 90th percentiles of BRI. When evidence of nonlinearity was detected, a two‐piecewise logistic regression model was subsequently applied to estimate associations below and above the inflection point. The log‐likelihood ratio test was used to compare the goodness of fit between the single‐line and two‐piecewise models.

Robustness of the associations was further evaluated through predefined subgroup analyses stratified by demographic (sex, age, education, marital status, residential area, employment), lifestyle (smoking, drinking), and health‐related variables (BMI category, depressive symptoms, hypertension, IADL disability). Interaction terms between BRI and each stratification variable were added to fully adjusted models to test for effect modification, assessed via likelihood ratio tests.

Exploratory analyses were additionally performed by cross‐classifying participants according to BMI (24.0 kg/m^2^ vs. ≥ 24.0 kg/m^2^) and BRI (low vs. high, dichotomized at the population median), yielding four groups, with the “low BMI + low BRI” group serving as the reference. Multivariable logistic regression models adjusting for all aforementioned covariates were used to estimate odds ratios (ORs) and 95% confidence intervals (CIs) for axial spinal pain across categories.

All statistical computations were carried out in R software (version 4.4.2; The R Foundation for Statistical Computing) in conjunction with EmpowerStats (X&Y Solutions, Inc., Boston, MA). Two‐tailed probability values below 0.05 were interpreted as statistically significant.

## 3. Results

### 3.1. Baseline Characteristics

Baseline characteristics of participants according to BRI quartiles are presented in Table 1. Height decreased progressively from Q1 to Q4 (160.8 ± 8.2 cm vs. 154.7 ± 8.4 cm; *p* < 0.001), whereas WC and BMI increased markedly (70.6 ± 12.9 cm to 97.1 ± 7.2 cm; *p* < 0.001, and 20.2 ± 2.4 kg/m^2^ to 27.1 ± 3.1 kg/m^2^; *p* < 0.001, respectively).

**TABLE 1:** Baseline characteristics by BRI quartiles (*n* = 10,689).

Characteristic	Q1 (*N* = 2672) (2.4 ± 0.9)	Q2 (*N* = 2672) (3.6 ± 0.2)	Q3 (*N* = 2672) (4.5 ± 0.3)	Q4 (*N* = 2673) (6.1 ± 1.0)	*p* value
Anthropometrics					
Height (cm)	160.8 ± 8.2	159.0 ± 8.3	157.7 ± 8.5	154.7 ± 8.4	< 0.001
Waist circumference (cm)	70.6 ± 12.9	81.5 ± 4.6	88.0 ± 5.1	97.1 ± 7.2	< 0.001
BMI (kg/m^2^)	20.2 ± 2.4	22.1 ± 2.0	24.2 ± 2.3	27.1 ± 3.1	< 0.001
Demographic factors					
Age (years)	62.5 ± 8.4	62.2 ± 8.5	62.4 ± 8.3	62.4 ± 8.2	0.681
Sex, *n* (%)					0.919
Male	1320 (49.4)	1315 (49.2)	1298 (48.6)	1302 (48.7)	
Female	1352 (50.6%)	1357 (50.8%)	1374 (51.4%)	1371 (51.3%)	
Education, *n* (%)					0.011
No formal education	1464 (54.8)	1440 (53.9)	1429 (53.5)	1476 (55.2)	
Elementary school	532 (19.9)	544 (20.4)	578 (21.6)	607 (22.7)	
Middle/high school	405 (15.2)	450 (16.8)	420 (15.7)	359 (13.4)	
College or higher	271 (10.1)	238 (8.9)	245 (9.2)	231 (8.6)	
Marital status, *n* (%)					0.619
Married	2143 (80.2)	2159 (80.8)	2181 (81.6)	2165 (81.0)	
Others	529 (19.8%)	513 (19.2%)	491 (18.4%)	508 (19.0%)	
Residential area, *n* (%)					0.119
Rural	1676 (62.7%)	1670 (62.5%)	1692 (63.3%)	1747 (65.4%)	
Urban	996 (37.3%)	1002 (37.5%)	980 (36.7%)	926 (34.6%)	
Has health insurance	2477 (93.0)	2504 (94.1)	2489 (93.7)	2496 (93.7)	0.461
Currently working, *n* (%)	1551 (58.9)	1626 (61.7)	1571 (59.7)	1637 (62.1)	0.045
Smoking status, *n* (%)					0.681
Never	1545 (57.8)	1558 (58.3)	1574 (58.9)	1558 (58.3)	
Former	282 (10.6)	247 (9.2)	249 (9.3)	250 (9.4)	
Current	845 (31.6)	867 (32.4)	849 (31.8)	865 (32.4)	
Drinking status, *n* (%)					0.062
Never	1555 (58.2)	1519 (56.8)	1593 (59.6)	1547 (57.9)	
Former	243 (9.1)	289 (10.8)	239 (8.9)	230 (8.6)	
Current	874 (32.7)	864 (32.3)	840 (31.4)	896 (33.5)	
Health status					
Depressive symptoms (CESD‐10), *n* (%)	7.0 (4.0–12.0)	8.0 (4.0–13.0)	7.0 (3.0–13.0)	8.0 (4.0–13.0)	0.490
Hypertension, *n* (%)	725 (27.2)	754 (28.4)	751 (28.2)	781 (29.3)	0.410
Diabetes, *n* (%)	187 (7.1)	186 (7.0)	185 (7.0)	159 (6.0)	0.338
IADL disability, *n* (%)					0.574
No limitation	2043 (76.5)	2009 (75.2)	2064 (77.2)	2038 (76.2)	
1 item limited	284 (10.6)	317 (11.9)	273 (10.2)	286 (10.7)	
≥ 2 items limited	345 (12.9)	346 (12.9)	335 (12.5)	349 (13.1)	
Primary outcome					
Axial spinal pain, *n* (%)	582 (21.8%)	601 (22.5%)	598 (22.4%)	677 (25.3%)	0.010

*Note:* Data are presented as mean ± standard deviation, median (interquartile range), or *n* (%). *p* values were derived from analysis of variance, the Kruskal–Wallis test, or *χ* ^2^ tests, as appropriate.

Abbreviations: BRI, body roundness index; BMI, body mass index; IADL, instrumental activities of daily living; CESD‐10, 10‐item Center for Epidemiologic Studies Depression Scale.

Demographic factors—including age (≈62 years across quartiles; *p* = 0.681), sex distribution (*p* = 0.919), marital status (*p* = 0.619), place of residence (*p* = 0.119), and health insurance coverage (*p* = 0.461) – remained comparable across quartiles. Educational attainment differed significantly (*p* = 0.011), with the proportion of participants lacking formal schooling highest in Q4 (55.2%). Current employment was more common in Q2 and Q4 (*p* = 0.045).

**FIGURE 2:**
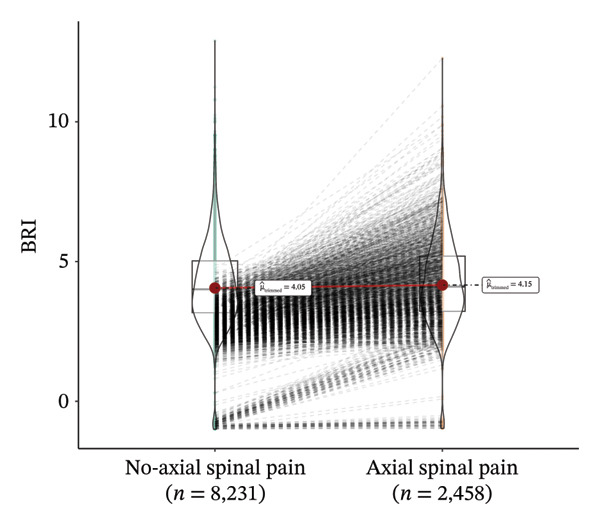
Paired comparison of body roundness index (BRI) in participants with and without axial spinal pain. Violin plots with box plots depict the distribution of BRI in the nonaxial spinal pain group (*n* = 8231) and the axial spinal pain group (*n* = 8231). Trimmed means are indicated by red points (4.05 vs. 4.15). Each thin black line connects matched individuals across groups, illustrating within‐pair differences in BRI. A predominance of upward‐sloping lines reflects a tendency towards higher BRI in participants with axial spinal pain.

Lifestyle factors (smoking, *p* = 0.681; drinking, *p* = 0.062) and health indicators (depressive symptoms, hypertension, diabetes, and IADL disability) did not differ significantly between quartiles. Notably, the prevalence of axial spinal pain increased from 21.8% in Q1 to 25.3% in Q4 (*p* = 0.010). As depicted in Figure 2, participants reporting axial spinal pain generally exhibited higher BRI values than those without pain, supporting a positive association between higher BRI and axial spinal pain prevalence.

### 3.2. Multivariable Regression Analysis of BRI and Axial Spinal Pain

Results of multivariable logistic regression models assessing the association between BRI and axial spinal pain are shown in Table 2. When analyzed as a continuous variable, BRI was significantly and positively associated with axial spinal pain across all models. In the crude model, each one‐unit increase in BRI was associated with a 4% higher odds of axial spinal pain (OR = 1.04, 95% CI 1.01–1.08; *p* = 0.004). This association remained significant after sequential adjustments for age and sex (Model 2: OR = 1.04, 95% CI 1.01–1.08; *p* = 0.005), socioeconomic and lifestyle factors (Model 3: OR = 1.04, 95% CI 1.01–1.07; *p* = 0.013), and additional health‐related covariates (fully adjusted Model 4: OR = 1.05, 95% CI 1.01–1.08; *p* = 0.007).

**TABLE 2:** Association between BRI and axial spinal pain: multivariable logistic regression analysis.

Exposure variable	Model 1 OR (95% CI)	*p* value	Model 2 OR (95% CI)	*p* value	Model 3 OR (95% CI)	*p* value	Model 4 OR (95% CI)	*p* value
BRI	1.04 (1.01, 1.08)	0.004	1.04 (1.01, 1.08)	0.005	1.04 (1.01, 1.07)	0.013	1.05 (1.01, 1.08)	0.007
Quartiles								
Q1	1.00 (reference)		1.00 (reference)		1.00 (reference)		1.00 (reference)	
Q2	1.04 (0.92, 1.19)	0.531	1.04 (0.92, 1.19)	0.536	1.04 (0.91, 1.18)	0.600	1.00 (0.87, 1.16)	0.968
Q3	1.04 (0.91, 1.18)	0.600	1.03 (0.91, 1.17)	0.642	1.03 (0.91, 1.18)	0.626	1.03 (0.90, 1.19)	0.651
Q4	1.22 (1.07, 1.38)	0.002	1.22 (1.07, 1.38)	0.003	1.20 (1.05, 1.36)	0.006	1.22 (1.06, 1.40)	0.005
*p* for trend		0.004		0.005		0.009		0.005

*Note:* Data are presented as odds ratio (OR) with 95% confidence interval (CI). Model 1: crude model. Model 2: adjusted for age and sex. Model 3: additionally adjusted for education, marital status, residential area, working status, smoking status, and drinking status. Model 4: additionally adjusted for health insurance, hypertension, diabetes, CESD‐10, and IADL disability. *p* for trend was calculated across the quartiles of body roundness index.

Abbreviations: BRI, body roundness index; IADL, instrumental activities of daily living; CESD‐10, 10‐item Center for Epidemiologic Studies Depression Scale.

When BRI was categorized into quartiles, no significant associations were observed for Q2 or Q3 compared with Q1. However, participants in Q4—the highest BRI quartile—had substantially higher odds of axial spinal pain (Model 1: OR = 1.22, 95% CI 1.07–1.38; *p* = 0.002; fully adjusted model: OR = 1.22, 95% CI 1.06–1.40; *p* = 0.005). A significant linear trend across quartiles was detected in all models (*p* for trend = 0.004–0.009).

Overall, these findings indicate that higher body roundness is independently associated with an increased likelihood of axial spinal pain, with the strongest effect observed among individuals in the highest BRI quartile.

### 3.3. Nonlinear Association and Threshold Effect Between BRI and Axial Spinal Pain

**FIGURE 3:**
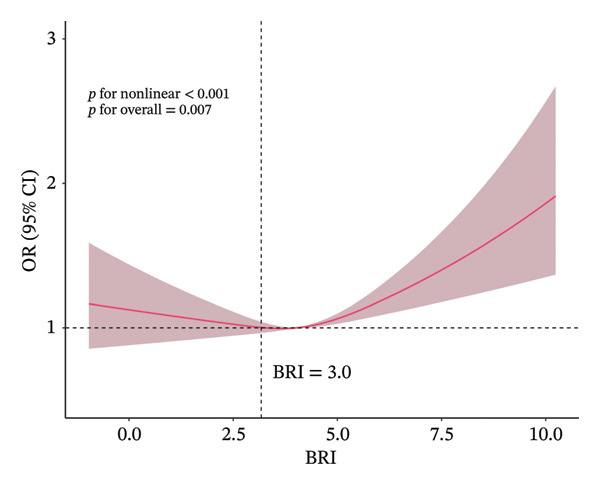
Nonlinear association between body roundness index (BRI) and axial spinal pain in the fully adjusted restricted cubic spline model. The fully adjusted model (controlling for demographic, lifestyle, and clinical covariates) identified BRI = 3.0 (vertical dashed line) as the threshold with odds ratio (OR) set to 1.0 (reference). The solid red line indicates the adjusted OR and the shaded area represents the 95% confidence interval. Below the threshold, the OR remained approximately stable; above it, the OR increased. Overall (*p* = 0.007) and nonlinearity (*p* < 0.001) tests were statistically significant.

The potential nonlinear association and threshold effect between BRI and axial spinal pain were evaluated using RCS modeling (Figure 3) and two‐piecewise logistic regression analysis (Table 3). After confirming the stability of the linear models, RCS analysis revealed a clear nonlinear relationship, with an inflection point observed at BRI = 3.

**TABLE 3:** Threshold effect of BRI on axial spinal pain.

Model	Nonadjusted model OR (95% CI)	*p* value	Fully‐adjusted model OR (95% CI)	*p* value
Inflection point (*K*)	3.0		3.0	
< *K*	0.93 (0.85, 1.01)	0.091	0.91 (0.83, 1.00)	0.044
> *K*	1.08 (1.04, 1.12)	< 0.0001	1.08 (1.04, 1.13)	< 0.0001
LRT test		0.006		0.002

*Note:* Data are presented as odds ratio (OR) with 95% confidence interval (CI). Fully‐adjusted model: adjusted for age, sex, education, marital status, residential area, working status, smoking status, drinking status, health insurance, hypertension, diabetes, CESD‐10, and IADL disability.

Abbreviations: BRI, body roundness index; IADL, instrumental activities of daily living; CESD‐10, 10‐item Center for Epidemiologic Studies Depression Scale.

Below this threshold, the crude model showed OR = 0.93 (95% CI 0.85–1.01; *p* = 0.091), and the fully adjusted model showed OR = 0.91 (95% CI 0.83–1.00; *p* = 0.044). Above the threshold, the crude model showed OR = 1.08 (95% CI 1.04–1.12; *p* < 0.0001), and the fully adjusted model showed OR = 1.08 (95% CI 1.04–1.13; *p* < 0.0001).

Log‐likelihood ratio tests comparing the segmented and single‐line models yielded *p* = 0.006 for the unadjusted and *p* = 0.002 for the fully adjusted analyses, indicating a superior fit of the two‐piecewise model. Overall, these results demonstrate a nonlinear relationship between BRI and axial spinal pain, with a stronger positive association emerging above the BRI threshold of approximately 3.

### 3.4. Subgroup Analysis

**FIGURE 4:**
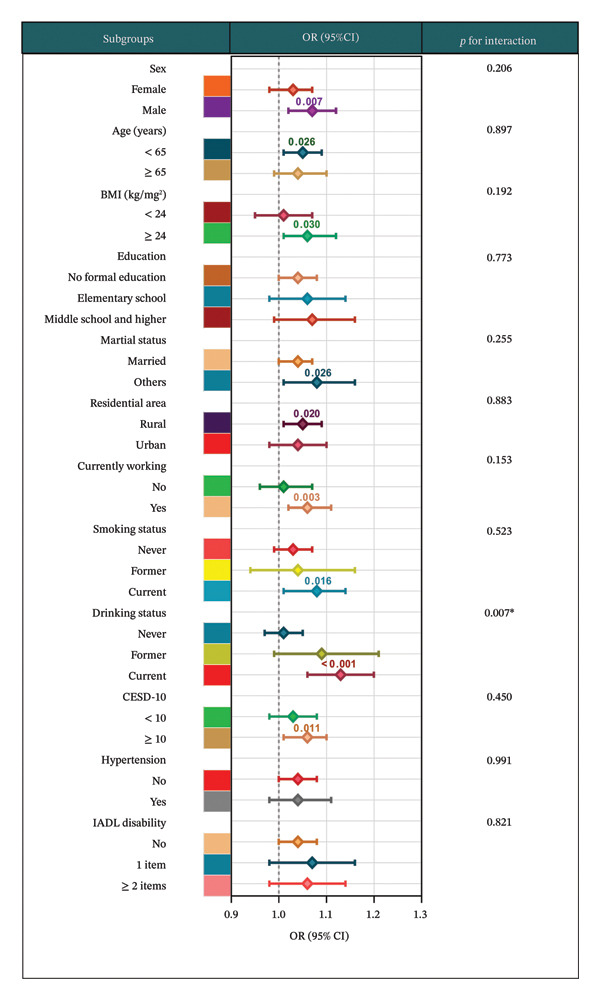
Subgroup analysis of associations with axial spinal pain. Odds ratios (OR) and 95% confidence intervals are shown for each subgroup. Analyses treated body roundness index (BRI) as a continuous variable and were based on fully adjusted models controlling for demographic, lifestyle, and clinical covariates. *p* values for interaction assess whether associations differ significantly across subgroups.

Subgroup analyses demonstrated that the positive association between BRI and axial spinal pain was generally consistent across most demographic and clinical subpopulations (Figure 4), with ORs ranging from 1.01 to 1.13. Overall, a higher BRI was associated with a modest yet stable increase in the likelihood of axial spinal pain.

Tests for interaction revealed no significant effect modification by the majority of examined factors, including sex (*p* for interaction = 0.206), age (*p* = 0.897), BMI (*p* = 0.192), educational attainment (*p* = 0.773), marital status (*p* = 0.255), residential area (*p* = 0.883), current employment status (*p* = 0.153), smoking status (*p* = 0.523), depressive symptoms (*p* = 0.450), hypertension (*p* = 0.991), and IADL disability (*p* = 0.821). These findings suggest that the association between BRI and axial spinal pain was robust and largely independent of these covariates. For example, the OR for BRI was 1.03 (95% CI 0.98–1.07) among females and 1.07 (95% CI 1.02–1.12) among males; 1.01 (95% CI 0.95–1.07) among participants with BMI < 24 kg/m^2^ and 1.06 (95% CI 1.01–1.12) among those with BMI ≥ 24 kg/m^2^.

**FIGURE 5:**
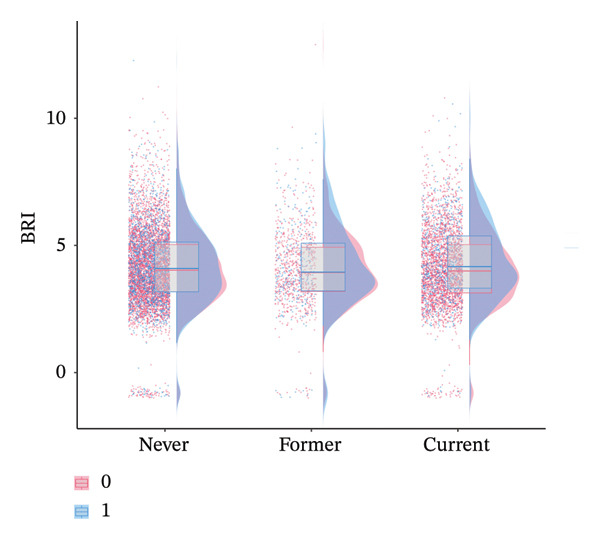
Raincloud plots of body roundness index (BRI) by drinking status and axial spinal pain groups. Raincloud plots combine kernel density distributions (cloud), box plots (central tendency and interquartile range), and raw data points (rain) to depict BRI among participants stratified by drinking status (never, former, and current) and axial spinal pain status (red = nonaxial spinal pain [0], blue = axial spinal pain [1]). Subgroup analysis demonstrated a significant positive interaction between drinking status and axial spinal pain, with the magnitude of BRI differences between pain groups varying across drinking categories.

A significant interaction was identified for drinking status (*p* for interaction = 0.007), indicating that alcohol consumption modified the association between BRI and axial spinal pain (Figure 5). Compared with participants who never drank (OR = 1.01, 95% CI 0.97–1.05), current drinkers exhibited a markedly stronger association (OR = 1.13, 95% CI 1.06–1.20), whereas former drinkers showed an intermediate effect (OR = 1.09, 95% CI 0.99–1.21). These observations highlight a potential amplifying effect of alcohol consumption on the relationship between body roundness and axial spinal pain.

### 3.5. Exploratory Analysis of the Association Between Combined BMI and BRI Categories and Axial Spinal Pain

In exploratory analyses modeling BRI as a continuous variable and stratifying by BMI, no significant association was observed in the low‐BMI group, whereas a positive association emerged among participants with higher BMI. Although the BMI × BRI interaction term did not reach statistical significance, the overall pattern suggested that general adiposity (BMI) and BRI may contribute independently and potentially additively to the risk of axial spinal pain.

To further explore this relationship, a combined BMI–BRI categorical analysis was performed. BMI was dichotomized using the Chinese adult threshold (< 24.0 kg/m^2^ vs. ≥ 24.0 kg/m^2^), and BRI was dichotomized at the population median, generating four exposure categories: low BMI + low BRI (reference), low BMI + high BRI, high BMI + low BRI, and high BMI + high BRI.

In fully adjusted logistic regression models controlling for demographic, socioeconomic, lifestyle, and health‐related factors, neither the low BMI + high BRI group (OR = 1.05, 95% CI 0.91–1.22; *p* = 0.497) nor the high BMI + low BRI group (OR = 1.05, 95% CI 0.83–1.32; *p* = 0.699) differed significantly from the reference group. In contrast, participants in the high BMI + high BRI category exhibited a significantly increased likelihood of axial spinal pain (OR = 1.16, 95% CI 1.04–1.30; *p* = 0.009).

**FIGURE 6:**
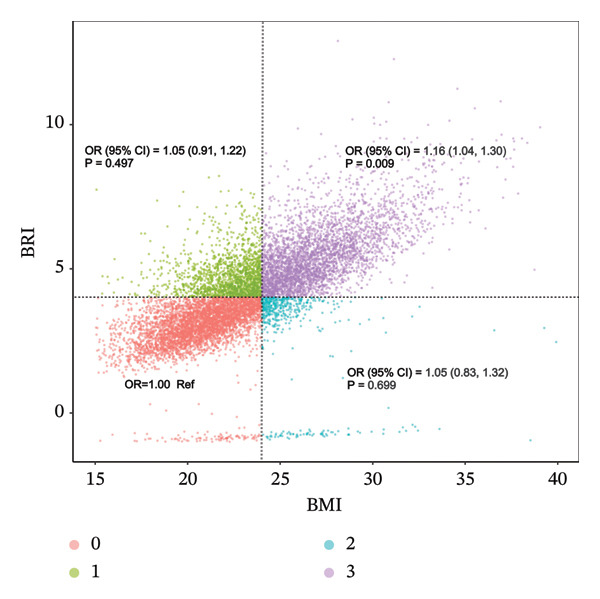
Combined categories of body mass index (BMI) and body roundness index (BRI) in relation to the odds of axial spinal pain. Participants were classified into four groups according to BMI (< 24 or ≥ 24 kg/m^2^) and BRI (low or high): 0 = low BMI + low BRI (reference), 1 = low BMI + high BRI, 2 = high BMI + low BRI, and 3 = high BMI + high BRI. Odds ratios (OR) and 95% confidence intervals were estimated using fully adjusted models controlling for demographic, lifestyle, and clinical covariates. A statistically significant positive association was observed only in Group 3 (OR = 1.16, 95% CI: 1.04–1.30, *p* = 0.009).

The consistent and graded pattern across categories (Figure 6) supports the hypothesis that elevated BMI and increased body roundness jointly contribute to spinal pain susceptibility, reflecting the additive burden of central and overall adiposity on spinal health.

### 3.6. Sensitivity Analyses

To assess the robustness of our primary findings, we conducted two sensitivity analyses. First, recognizing that physical activity (PA) is a potential confounder in the association between adiposity and spinal pain, we further adjusted our fully adjusted model for PA level among participants with complete PA data (*n* = 4478). Despite a substantial reduction in sample size due to missing PA data, the association between BRI (continuous) and axial spinal pain remained significant (OR = 1.05, 95% CI 1.01–1.08; *p* = 0.007) (Supporting Table S1), consistent with the main analysis. This suggests that our results are robust to additional adjustment for PA. Second, to address the generalizability to a broader middle‐aged population, we expanded our analytic sample to include participants aged 45–49 years (total *n* = 13,182). The fully adjusted model showed a similar positive association between BRI and axial spinal pain (OR = 1.07, 95% CI 1.04–1.10; *p* < 0.001) (Supporting Table S2). These findings indicate that the exclusion of the 45–49 age group did not materially affect our conclusions.

## 4. Discussion

In this nationally representative, cross‐sectional study of middle‐aged and older adults, a higher BRI was positively associated with the presence of axial spinal pain. This relationship remained significant after adjustment for demographic, socioeconomic, lifestyle, and health‐related covariates and was consistent across most subgroups. A clear nonlinear dose–response pattern was identified, characterized by a steeper increase in risk above a BRI value of approximately 3.0. Furthermore, the observed interaction with alcohol consumption suggests that drinking status may modify the association between BRI and spinal pain. To the best of our knowledge, this is the first study to comprehensively evaluate the link between body roundness and axial spinal pain in this population. In addition, exploratory analyses combining BMI and BRI revealed that individuals with elevated values in both indices were more likely to experience axial spinal pain, underscoring the potential additive influence of overall and central adiposity.

### 4.1. Comparison With Previous Studies

Our findings are in line with prior epidemiological evidence linking greater adiposity to increased risk of musculoskeletal pain, particularly low back or waist pain [18–20]. While BMI reflects total body mass, BRI incorporates height and WC, offering a more refined estimation of central adiposity [15]. Most previous research on BRI has focused on metabolic and cardiovascular outcomes [21–23], whereas musculoskeletal outcomes among older adults have been comparatively underexplored. Age‐related factors—such as progressive muscle loss, altered fat distribution, and spinal degeneration—may amplify these associations [24]. Earlier studies have also reported that individuals with both elevated BMI and WC above the central‐obesity threshold exhibited stronger associations with low back pain compared with elevated BMI alone [25–26]. Building on these observations, our cross‐sectional analysis found that concurrent elevations in BMI and BRI were more strongly associated with axial spinal pain than either index individually, underscoring the potential utility of BRI for musculoskeletal health assessment beyond its established cardiometabolic applications. Nevertheless, given the cross‐sectional design of this study, causality cannot be inferred, and longitudinal research is warranted to clarify the temporal and mechanistic pathways underlying these relationships.

### 4.2. Potential Mechanisms

Several plausible mechanisms may underlie the observed associations, though causal inference is limited by the cross‐sectional design of this study.

From a mechanical perspective, central adiposity can increase spinal loading, alter alignment, and elevate stress on intervertebral discs and facet joints [27–29]. From a biological standpoint, metabolically active visceral fat secretes proinflammatory cytokines (e.g., interleukin‐6 and tumor necrosis factor‐α) that may sensitize nociceptors and amplify pain perception [30, 31]. Additionally, abdominal fat accumulation has been linked to fatty infiltration of paraspinal muscles (e.g., multifidus), potentially diminishing spinal stability [32, 33].

The statistically significant interaction with alcohol consumption, despite its modest effect size, raises the possibility that drinking may exacerbate obesity‐related systemic inflammation [34]. This interpretation remains speculative and requires validation in longitudinal studies incorporating inflammatory biomarker profiling.

Taken together, our findings support the hypothesis that central adiposity exhibits a threshold effect (BRI ≈ 3.0), beyond which its impact becomes clinically relevant. The greatest impact appears to arise from the additive influences of general and central adiposity, through mechanical [18] and metabolic‐inflammatory pathways [25, 35]. Confirmation of these mechanisms and clarification of their temporal sequence will require prospective longitudinal investigation.

### 4.3. Clinical Implications

Although causal inference is limited by the cross‐sectional design, our findings suggest that the BRI may serve as a practical and informative anthropometric measure for evaluating central adiposity in relation to axial spinal pain among middle‐aged and older adults. Incorporating BRI together with conventional metrics such as BMI and WC could enhance clinical assessment of body composition relevant to spinal health and help guide individualized management strategies addressing both overall and central adiposity. The observed correlation between BRI and alcohol consumption, as well as the coexistence of elevated BMI and BRI, indicates that modifiable lifestyle factors may contribute to variations in spinal pain presentation, highlighting the potential value of comprehensive behavioral interventions. At a broader public‐health level, applying BRI in community screening and elderly care programs provides a simple and reproducible approach to identifying central adiposity patterns associated with spinal pain, thereby supporting the design of targeted health‐promotion and prevention initiatives.

### 4.4. Strengths and Limitations

This study benefits from a nationally representative cohort of middle‐aged and older Chinese adults, enabling comprehensive examination of central adiposity and axial spinal pain across diverse demographic and geographic groups. Standardized protocols, calibrated instruments, and rigorous eligibility criteria ensured high‐quality data collection and minimized selection bias. Extensive adjustments for demographic, socioeconomic, lifestyle, and health‐related factors—supported by subgroup, interaction, and combined‐effect analyses with sensitivity verification—enhanced the internal validity and robustness of the results. Incorporating the BRI alongside BMI provides methodological novelty, extending musculoskeletal epidemiology beyond traditional anthropometric measures.

Several potential limitations should also be acknowledged. First, the exclusively Chinese cohort may limit the generalizability of our findings to other ethnic or cultural contexts. Second, the cross‐sectional design constrains causal inference, and although higher BRI was associated with a greater likelihood of spinal pain, reverse causality cannot be excluded (e.g., chronic pain reducing activity and promoting central fat accumulation). Third, residual confounding from unmeasured variables remains possible despite extensive adjustment. Fourth, axial spinal pain was defined using self‐reported back or waist pain, which may not capture the full clinical heterogeneity of spinal disorders; moreover, self‐report introduces recall bias and may compromise the precision of symptom ascertainment. The absence of pain‐severity assessment further restricts evaluation of disease burden and potential dose‐response patterns. Fifth,alcohol‐consumption data lacked granularity regarding quantity and frequency, which may attenuate the precision of interaction estimates. Future multicenter longitudinal studies across diverse populations are warranted to validate these findings, clarify causal pathways, and assess the clinical utility of BRI in predicting spinal pain risk. Finally, an important limitation of this study pertains to the assessment of pain severity. The CHARLS questionnaire did not collect site‐specific pain severity information (e.g., severity of low back pain specifically). Therefore, we were unable to examine whether BRI exhibits a dose‐response relationship with the severity of axial spinal pain. We acknowledge this as an inherent limitation of the dataset and encourage future research to employ site‐specific pain severity instruments to better elucidate this relationship.

## 5. Conclusion

In this nationally representative cross‐sectional study of middle‐aged and older Chinese adults, a higher BRI was independently associated with increased odds of axial spinal pain, with the strength of association varying by BRI level and lifestyle factors. The apparent additive influence of central and general adiposity highlights the potential value of incorporating BRI into musculoskeletal risk assessment alongside conventional anthropometric indices. Given the observational nature of the study, prospective longitudinal research is needed to establish temporal relationships, elucidate underlying mechanisms, and determine whether integrating BRI can enhance strategies for the prevention and management of axial spinal pain.

## Funding

This work was supported by the Huzhou Municipal Science and Technology Bureau (Grant Number 2025GY105).

## Ethics Statement

Ethical approval for the CHARLS 2011 baseline survey was granted by the Peking University Biomedical Ethics Review Committee (IRB00001052‐11014). All participants provided written informed consent before taking part in the study, which was conducted in strict compliance with ethical standards and relevant regulations.

## Consent

Please see the Ethics Statement.

## Conflicts of Interest

The authors declare no conflicts of interest.

## Supporting Information

Additional supporting information can be found online in the Supporting Information section.

## Supporting information


**Supporting Information 1** Supporting Table S1: Sensitivity analysis: association between BRI and axial spinal pain after additional adjustment for physical activity in participants with complete PA data (*n* = 4478).

**Supporting Information 2** Supporting Table S2: Sensitivity analysis: association between BRI and axial spinal pain after expanding the age range to include participants aged 45 years and older (*n* = 13,182).

## Data Availability

The data supporting the findings of this study are publicly available from the China Health and Retirement Longitudinal Study (CHARLS) repository, which can be accessed at: https://charls.pku.edu.cn/.
