# Supplementary material for: Nonlinear Association Between Body Roundness Index and Axial Spinal Pain in Middle‐Aged and Older Chinese Adults: A Nationwide Cross‐Sectional Study
Source: Pain Res Manag. 2026 Apr 1;2026:3187891. doi: 10.1155/prm/3187891 (PMC13042353; doi:10.1155/prm/3187891)
Supplement: Supplementary file 1 — Supporting Information 1 Supporting Table S1: Sensitivity analysis: association between BRI and axial spinal pain after additional adjustment for physical activity in participants with complete PA data (n = 4478). [file PRM-2026-3187891-s001.docx]

****Supplementary Table S1. Sensitivity Analysis: Association Between BRI and Axial Spinal Pain After Additional Adjustment for Physical Activity in Participants with Complete PA Data (n = 4,478)****

| **Exposure variable** | **Model 1**  **OR (95%CI)** | **P value** | **Model 2**  **OR (95%CI)** | **P value** | **Model 3**  **OR (95%CI)** | **P value** |
| --- | --- | --- | --- | --- | --- | --- |
| **BRI** | 1.04 (1.01, 1.08) | 0.004 | 1.05 (1.01, 1.08) | 0.007 | 1.05 (1.01, 1.08) | 0.007 |
| **Quartiles** |  |  |  |  |  |  |
| Q1 | 1.00 (Reference) |  | 1.00 (Reference) |  |  |  |
| Q2 | 1.04 (0.92, 1.19) | 0.531 | 1.00 (0.87, 1.16) | 0.968 | 1.00 (0.87, 1.15) | 0.993 |
| Q3 | 1.04 (0.91, 1.18) | 0.600 | 1.03 (0.90, 1.19) | 0.651 | 1.04 (0.90, 1.19) | 0.638 |
| Q4 | 1.22 (1.07, 1.38) | 0.002 | 1.22 (1.06, 1.40) | 0.005 | 1.22 (1.06, 1.40) | 0.006 |
| **P for trend** |  | 0.004 |  | 0.005 |  | 0.005 |

Data are presented as odds ratio (OR) with 95% confidence interval (CI).

Model 1: Crude model.

Model 2: Fully-Adjusted Model, adjusted for age, sex, education, marital status, residential area, working status, smoking status, drinking status, health insurance, hypertension, diabetes, CESD-10, and IADL disability.

Model 3: Adjusted for all covariates in Model 2 plus physical activity.

P for trend was calculated across the quartiles of body roundness index.

*Abbreviation: BRI, Body Roundness Index; IADL, Instrumental Activities of Daily Living; CESD-10, 10-item Center for Epidemiologic Studies Depression Scale.*
