# Supplementary material for: Nonlinear Association Between Body Roundness Index and Axial Spinal Pain in Middle‐Aged and Older Chinese Adults: A Nationwide Cross‐Sectional Study
Source: Pain Res Manag. 2026 Apr 1;2026:3187891. doi: 10.1155/prm/3187891 (PMC13042353; doi:10.1155/prm/3187891)
Supplement: Supplementary file 2 — Supporting Information 2 Supporting Table S2: Sensitivity analysis: association between BRI and axial spinal pain after expanding the age range to include participants aged 45 years and older (n = 13,182). [file PRM-2026-3187891-s002.docx]

****Supplementary Table S2. Sensitivity Analysis: Association Between BRI and Axial Spinal Pain After Expanding the Age Range to Include Participants Aged 45 Years and Older (n = 13,182)****

| **Exposure variable** | **Crude Model**  **OR (95% CI)** | **P value** | **Fully-Adjusted Model**  **OR (95%CI)** | **P value** |
| --- | --- | --- | --- | --- |
| **BRI** | 1.06 (1.03, 1.09) | <0.001 | 1.07 (1.04, 1.10) | <0.001 |
| **Quartiles** |  |  |  |  |
| **Q1** | 1.00 (Reference) |  | 1.00 (Reference) |  |
| **Q2** | 1.03 (0.92, 1.16) | 0.581 | 1.02 (0.90, 1.15) | 0.782 |
| **Q3** | 1.06 (0.94, 1.20) | 0.342 | 1.05 (0.93, 1.19) | 0.424 |
| **Q4** | 1.25 (1.11, 1.41) | <0.001 | 1.24 (1.09, 1.40) | 0.001 |
| **P for trend** |  | 0.002 |  | 0.003 |

Data are presented as odds ratio (OR) with 95% confidence interval (CI).

Fully-Adjusted Model: Adjusted for age, sex, education, marital status, residential area, working status, smoking status, drinking status, health insurance, hypertension, diabetes, CESD-10, and IADL disability.

*Abbreviation: BRI, Body Roundness Index; IADL, Instrumental Activities of Daily Living; CESD-10, 10-item Center for Epidemiologic Studies Depression Scale.*

P for trend was calculated across the quartiles of body roundness index.

*Abbreviation: BRI, Body Roundness Index; IADL, Instrumental Activities of Daily Living; CESD-10, 10-item Center for Epidemiologic Studies Depression Scale.*
